# Colistin‐resistant *Entero* *bacter kobei* carrying *mcr‐9.1* and *bla*_CTX‐M‐15_ infecting a critically endangered franciscana dolphin (*Pontoporia blainvillei*), Brazil

**DOI:** 10.1111/tbed.13980

**Published:** 2021-05-06

**Authors:** Danny Fuentes‐Castillo, Fábio P. Sellera, Daphne W. Goldberg, Herrison Fontana, Fernanda Esposito, Brenda Cardoso, Joana Ikeda, Anneliese Kyllar, José L. Catão‐Dias, Nilton Lincopan

**Affiliations:** ^1^ Department of Pathology School of Veterinary Medicine and Animal Sciences University of São Paulo São Paulo Brazil; ^2^ One Health Brazilian Resistance Project (OneBR) São Paulo Brazil; ^3^ Department of Internal Medicine School of Veterinary Medicine and Animal Science University of São Paulo São Paulo Brazil; ^4^ Econservation/Santos Basin Beach Monitoring Project Rio de Janeiro Brazil; ^5^ Department of Clinical Analysis School of Pharmacy University of São Paulo São Paulo Brazil; ^6^ Department of Microbiology Instituto de Ciências Biomédicas Universidade de São Paulo São Paulo Brazil; ^7^ Laboratory of Aquatic Mammals and Bioindicators: Profa Izabel M. G. do N. Gurgel’ (MAQUA) Faculty of Oceanography Rio de Janeiro State University Rio de Janeiro Brazil; ^8^ CTA/Santos Basin Beach Monitoring Project Rio de Janeiro Brazil

**Keywords:** Enterobacterales, MCR, multidrug‐resistant, One Health, polymyxin, Wildlife

## Abstract

The emergence of mobile *mcr* genes mediating resistance to colistin is a critical public health issue that has hindered the treatment of serious infections caused by multidrug‐resistant pathogens in humans and other animals. We report the emergence of the *mcr‐9.1* gene in a polymyxin‐resistant extended‐spectrum β‐lactamase (ESBL)‐producing *Enterobacter kobei* infecting a free‐living franciscana dolphin (*Pontoporia blainvillei*), threatened with extinction in South America. Genomic analysis confirmed the presence of genes conferring resistance to clinically relevant β‐lactam [*bla*_CTX‐M‐15_, *bla*_ACT‐9_, *bla*_OXA‐1_ and *bla*_TEM‐1B_], aminoglycoside [*aac(3)‐IIa*, *aadA1*, *aph(3'')‐Ib* and *aph(6)‐Id*], trimethoprim [*dfrA14*], tetracycline [*tetA*], quinolone [*aac(6')‐Ib‐cr* and *qnrB1*], fosfomycin [*fosA*], sulphonamide [*sul2*] and phenicol [*catA1* and *catB3*] antibiotics. The identification of *mcr‐9.1* in a CTX‐M‐15‐producing pathogen infecting a critically endangered animal is of serious concern, which should be interpreted as a sign of further spread of critical priority pathogens and their resistance genes in threatened ecosystems.

## 1 INTRODUCTION

The global emergence and rapid dissemination of mobile phosphoethanolamine transferase *mcr* genes, responsible for transferable colistin resistance in Enterobacterales, is a public health concern (El‐Sayed Ahmed et al., 2020; Wang, Liu, et al., 2020). In this regard, since the first report of the *mcr‐1* gene, in 2015, novel alleles including *mcr‐2*, *mcr‐3*, *mcr‐4*, *mcr‐5*, *mcr‐6*, *mcr‐7*, *mcr‐8*, *mcr‐9* and *mcr‐10* have been globally identified (El‐Sayed Ahmed et al., 2020; Li et al., 2020; Ling et al., 2020; Liu et al., 2016; Wang, Feng, et al., 2020). Worryingly, the occurrence of *mcr* genes has been documented in critical priority extended‐spectrum β‐lactamase (ESBL)‐producing pathogens, mostly isolated from humans and food‐producing animals (El‐Sayed Ahmed et al., 2020; Liu et al., 2016; Wang, Liu, et al., 2020).

The franciscana dolphin (*Pontoporia blainvillei*) is considered the most threatened small cetacean in the south‐western Atlantic Ocean, which includes the coasts of Brazil, Uruguay and Argentina (Sucunza et al., 2018). Due to their coastal habits, these animals have been frequently exposed to different degrees of anthropogenic impacts, including fisheries by catch and habitat degradation (Sucunza et al., 2018). Consequently, this species is currently listed as vulnerable to extinction by the International Union for Conservation of Nature (Cunha et al., 2014; Zerbini et al., 2017), and as critically endangered by the Red Book of Threatened Species of Fauna, Brazil (ICMBio, 2018).

In this study, we report the emergence of *mcr‐9.1* in an ESBL‐producing *E. kobei* infecting a free‐living franciscana dolphin in Brazil. Additionally, an epidemiological landscape of global distribution of MCR‐9‐producing Enterobacterales circulating at human‐animal interface is presented.

## 2 MATERIALS AND METHODS

In December 2019, a female neonate franciscana was found stranded alive in Mambucaba Beach, in Angra dos Reis (−23.027184, −44.518130), located in the Southern coast of Rio de Janeiro state, Brazil (Figure S1). The animal was rescued by the staff of the Santos Basin Beach Monitoring Project (PMP‐BS), presenting excoriations on the head and with part of the umbilical cord still present. The dolphin was closely monitored, receiving intensive care and bottle‐feeding with a special dolphin formula every 3 hr. However, after 11 hr in captivity, the animal began to exhibit clinical signs of shock leading to death. In order to determine the main causes of death, necropsy was performed, where histopathological analysis of fixed lung tissue revealed severe pneumonia. Additionally, bacteriological culture of respiratory exudate collected through the spiracle was positive for Gram‐negative bacilli.

Antimicrobial susceptibility testing was performed by the disc diffusion method (CLSI, 2020), including amoxicillin/clavulanic acid, aztreonam, cefotaxime, ceftriaxone, cefepime, cefoxitin, ceftiofur, ciprofloxacin, enrofloxacin, chloramphenicol, amikacin, gentamicin, ertapenem, imipenem, meropenem, sulfamethoxazole/trimethoprim and tetracycline. In addition, colistin susceptibility testing was performed by broth microdilution method (EUCAST, 2020). The minimum inhibitory concentration (MIC) for fosfomycin was determined by using the agar dilution method (CLSI, 2020). ESBL production was screened by the double‐disc synergy test (DDST; Jarlier et al., 1988). *Escherichia coli* ATCC 25,922 was used as control strain. Bacterial conjugation for the *mcr‐9.1*‐positive *E*. *kobei* isolate was done in a liquid and solid mating‐out assay (Lampkowska et al., 2008), using the azide‐resistant *E. coli* C600 as recipient.

Genomic DNA was extracted and used to construct a paired‐end library, which was sequenced using the NextSeq 550 platform (Illumina), using paired‐end reads (150 bp). De novo genome assembly and contig annotation was carried out using CLC Genomics Workbench 12.0.3. Prediction of bacterial species, resistome and plasmidome was performed using fast K‐mer algorithm KmerFinder 3.2 (Larsen et al., 2014), ResFinder 3.2 (Zankari et al., 2012) and PlasmidFinder 2.1 (Carattoli et al., 2014) databases, respectively (http://www.genomicepidemiology.org/).

## 3 RESULTS AND DISCUSSION

The Gram‐negative bacilli were identified as belonging to the *Enterobacter cloacae* complex (E11R strain) by using matrix‐assisted laser desorption/ionization time‐of‐flight mass spectrometry (MALDI‐TOF). The E11R strain displayed a multidrug‐resistant (MDR) profile (Magiorakos et al., 2012) to amoxicillin/clavulanic acid, aztreonam, cefotaxime, ceftriaxone, cefepime, cefoxitin, ceftiofur, ciprofloxacin, enrofloxacin, chloramphenicol, fosfomycin (MIC, >1,024 µg/ml), gentamicin, sulfamethoxazole/trimethoprim and tetracycline, remaining susceptible to ertapenem, imipenem, meropenem and amikacin. Furthermore, E11R strain exhibited resistance to colistin (MIC, 16 µg/ml), whereas ESBL production was detected by the DDST.

**FIGURE 1:**
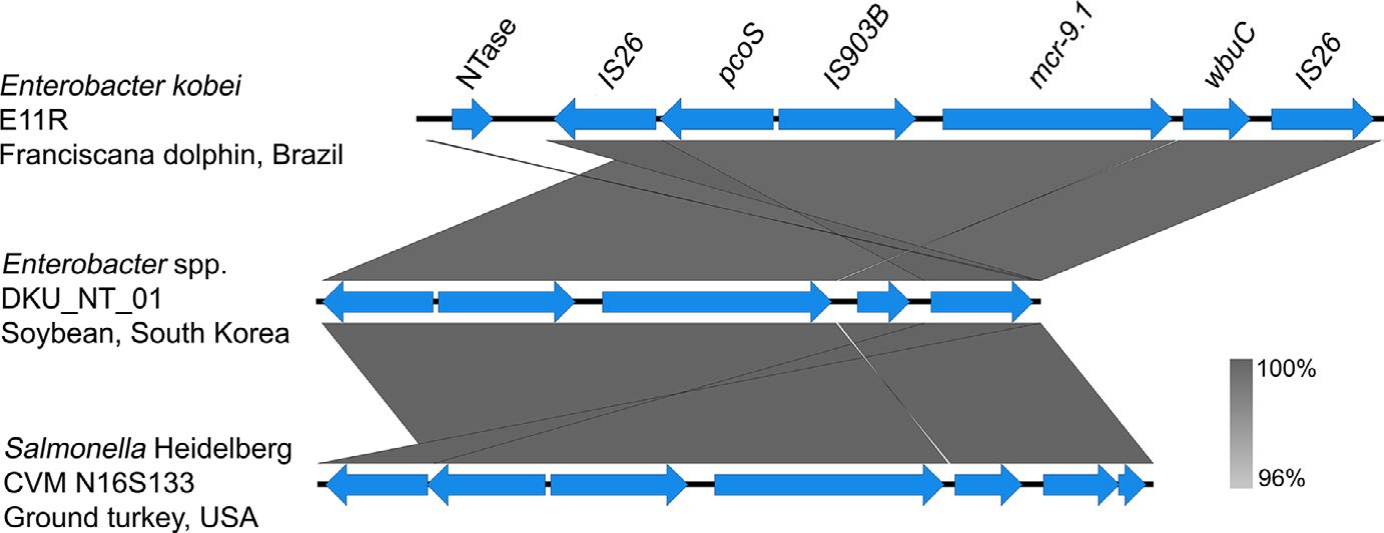
Genetic context of the *mcr‐9.1* gene in the colistin‐resistant *Enterobacter kobei* strain E11R. IS*903B* and IS*26* elements were found upstream and downstream of *mcr‐9.1* in a similar way that in MCR‐9‐producing *Enterobacter* spp. DKU_NT_01 strain (GenBank accession number: CP021137.1) isolated from soybean in South Korea; *Salmonella* Saintpaul [CVM N16S133 (CP049986.1), NY‐N14748 (CP048926.1), CVM N40391 (CP049983.1) and CVM N52030 (CP049981.1)], *S*. Heidelberg [CVM N16S321 (CP049313.1), CVM N58631 (CP049307.1) and CVMN53023 (CP049310.1)], and *S*. Albany [CVM N18S2238 (CP049312)] strains isolated from ground turkey; *S*. Johannesburg [CVM N58011 (CP049309)] strain isolated from chicken breast; and *Escherichia coli* [CVM N18EC0432 (CP048293.1)] strain isolated from chicken wings, in the United States of America (Tyson et al., 2020)

Genomic analysis identified the E11R strain as *E*. *kobei*, confirming a wide resistome, with genes conferring resistance to colistin [*mcr‐9.1*], β‐lactams [*bla*_CTX‐M‐15_, *bla*_ACT‐9_, *bla*_OXA‐1_ and *bla*_TEM‐1B_], aminoglycosides [*aac(3)‐IIa*, *aadA1*, *aph(3'')‐Ib* and *aph(6)‐Id*], trimethoprim [*dfrA14*], tetracycline [*tetA*], quinolones [*aac(6')‐Ib‐cr* and *qnrB1*], fosfomycin [*fosA*], sulphonamide [*sul2*] and phenicols [*catA1* and *catB3*]. IncHI2 and IncHI2A replicons were detected, and analysis of the genetic environment confirmed that *mcr‐9.1* was flanked by the insertion sequences IS*903B* and IS*26*, as previously reported (Figure 1; Kieffer et al., 2019; Lin et al., 2020; Tyson et al., 2020; Yuan et al., 2019). The region upstream of *mcr‐9*.*1* in *E*. *kobei* E11R strain included nucleotidyltransferase (NTase, transferase enzyme), *IS26*, *pcoS* (encoding a two‐component sensor histidine kinase) and IS*903B*. On the other hand, the region downstream of *mcr‐9.1* included *wbuC* (encoding a cupin fold metalloprotein), but no genes encoding the two‐component system *qseC‐qseB*, which has been associated with the expression of *mcr‐9* in other Enterobacterales (Kananizadeh et al., 2020; Kieffer et al., 2019). Conjugation attempts to evaluate the transferability of the *mcr‐9.1* gene were unsuccessful. In this regard, absence of the *qseC‐qseB* genes in the *E*. *kobei* E11R strain could be associated with the unsuccessful selection of transconjugants in agar plates supplemented with colistin (1 mg/ml) (Tyson et al., 2020).

In recent years, colistin has been used as a last‐resort for the treatment of infections caused by multidrug‐resistant and/or carbapenem‐resistant Gram‐negative bacteria (El‐Sayed Ahmed et al., 2020). However, the previous and extensive use of colistin in production animals, as a growth promoter or for prophylaxis, has been recognized as a responsible factor for the emergence and the rapid dissemination of mobile colistin resistance (*mcr*) genes (Rhouma et al., 2016). In this respect, since the detection of *mcr‐1*, nine additional *mcr* homologues have been described, with several gene variants occurring worldwide (El‐Sayed Ahmed et al., 2020; Wang, Liu, et al., 2020).

**FIGURE 2:**
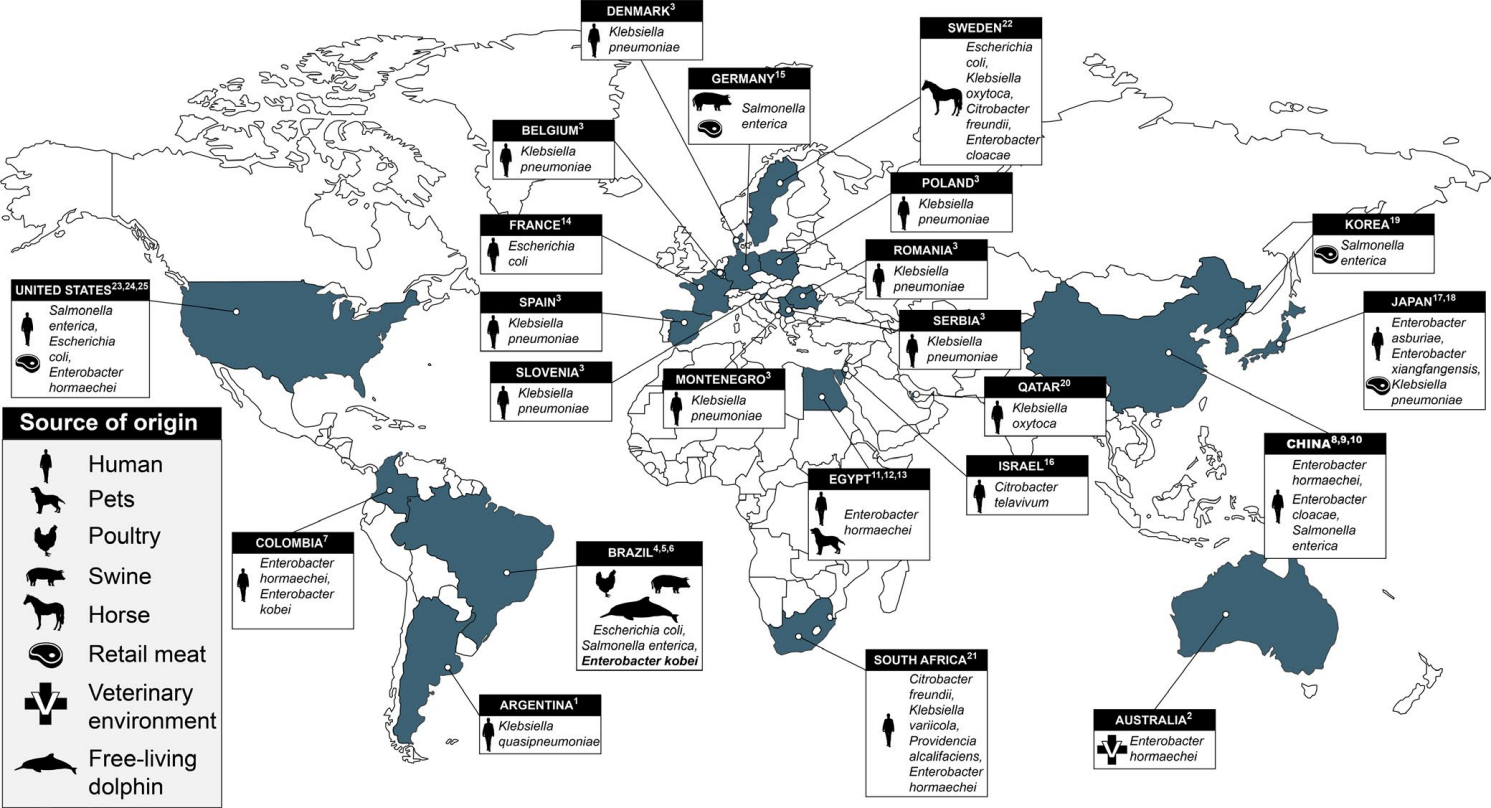
Global distribution of MCR‐9‐positive Enterobacterales. The occurrence of MCR‐9‐producing Enterobacterales (*i.e*. *Citrobacter freundii*, *Citrobacter telavivum, Enterobacter asburiae, Enterobacter cloacae*, *Enterobacter hormaechei*, *Enterobacter kobei*, *Enterobacter xiangfangensis Escherichia coli*, *Klebsiella oxytoca*, *Klebsiella pneumoniae*, *Klebsiella quasipneumoniae*, *Klebsiella variicola*, *Providencia alcalifaciens* and *Salmonella enterica*) has been reported in Argentina (1, Faccone et al., 2020), Australia (2, Kamathewatta et al., 2020), Belgium (3, Wang, Liu, et al., 2020), Brazil (4, Saidenberg et al., 2020; 5, Leite et al., 2020; 6, This study), Colombia (7, Rada et al., 2020), China (8, Yuan et al., 2019; 9, Lin et al., 2020; 10, Pan et al., 2020), Denmark (3, Wang, Liu, et al., 2020), Egypt (11, Khalifa, Oreiby, 2020; 12, Sadek et al., 2020; 13, Soliman et al., 2020), France (14, Kieffer et al., 2019), Germany (15, Borowiak et al., 2020), Israel (16, Ribeiro et al., 2020), Japan (17, Kananizadeh et al., 2020; 18, Khalifa, Soliman, et al., 2020), Korea (19, Cha et al., 2020), Montenegro (3, Wang, Liu, et al., 2020), Poland (3, Wang, Liu, et al., 2020), Qatar (20, Tsui et al., 2020), Romania (3, Wang, Liu, et al., 2020), Serbia (3, Wang, Liu, et al., 2020), Slovenia (3, Wang, Liu, et al., 2020), South Africa (21, Sekyere et al., 2020), Spain (3, Wang, Liu, et al., 2020), Sweden (22, Börjesson et al., 2020) and the United States of America (23, Carrol et al, 2019; 24, Chavda et al., 2019; 25, Tyson et al., 2020), from human and non‐human sources

The *mcr‐9.1* allele was identified for the first time in *Salmonella* Typhimurium isolated from a human patient (Carroll et al., 2019) and currently has been reported worldwide with a rapid dissemination among Enterobacterales from human, food, poultry, pets, swine and horse samples (Figure 2). Recently, two novel variants, *mcr‐9.2* and *mcr‐9.3*, have been identified in *Enterobacter hormaechei* subsp. *xiangfangensis* (GenBank accession number: MN164032.1) and *Klebsiella pneumoniae* (GenBank accession number: MT505326.1) isolates, respectively.

In this study, we report the emergence of *mcr‐9.1* in an ESBL‐producing *E. kobei* isolated from an infected free‐living franciscana dolphin, a species critically endangered by anthropogenic activities in Brazil (Cunha et al., 2014; ICMBio, 2018). The occurrence of CTX‐M‐15‐producing *E*. *coli* has been reported in captive dolphins (Manageiro et al., 2015), and now, we demonstrated that this type of pathogen can also threaten free‐living dolphins, which may have serious implications for wild populations and associated ecosystems.

The environmental dissemination of critical priority pathogens has been considered a serious threat to ecosystem maintenance (de Carvalho et al., 2020; Sevilla et al., 2020; Founou et al., 2019; Sekyere et al., 2020). This issue considered another form of environmental pollution (Guenther et al., 2011), could also substantially increase the risk for marine populations acquire such bacteria (Power et al., 2016). Specifically in Brazilian coast, the occurrence of MCR‐type, ESBL‐ and/or carbapenemase‐producing bacteria has been documented in recreational waters (Campana et al., 2017; Fernandes et al., 2017; Paschoal et al., 2017; Sellera, Fernandes, Moura, et al., 2017), beach sand samples (Furlan et al., 2020), mangrove waters (Sacramento et al., 2018), and marine hosts (Goldberg et al., 2019; Sellera et al., 2018; Sellera, Fernandes, Sartori, et al., 2017). In this way, considering the One Health perspective, environment and wild animals are also acting as bioindicators for clinically important antibiotic‐resistant pathogens that can seriously affect human communities related with these ecosystems (McEwen & Collignon, 2018; White & Hughes, 2019).

In summary, we report the emergence of MCR‐9‐producing bacteria in marine wildlife. Considering that oceanic environments and human and animal health are strictly connected, the dissemination of clinically important MDR pathogens in marine ecosystems must be viewed as serious One Health problem. Finally, since multidrug‐resistant pathogens have begun to be associated with fatal cases of infections in endangered animals (Fuentes‐Castillo et al., 2020), continued surveillance of MCR‐ and ESBL‐producing bacteria in marine ecosystems should be globally performed for a better comprehension of the transmission pathways and clinical impacts on marine wildlife.

## CONFLICT OF INTERESTS

No potential conflict of interest was reported by the authors.

## ETHICAL APPROVAL

The authors confirm that the ethical policies of the journal, as noted on the journal's author guidelines page, have been adhered to. No ethical approval was required for this specific study. The licences and research permit for monitoring programme and the biological sampling were issued by the Brazilian government (IBAMA‐ABIO 624/2015); all animal handling procedures and protocols followed the required ethics and welfare practices.

## Supporting information

Supplementary Material

## Data Availability

The whole genome nucleotide sequence of the *E. kobei* E11R isolate is available in the GenBank database under accession number PRJNA615090. Additionally, genomic data of *E. kobe* E11R strain will be available in the OneBR platform (http://onehealthbr.com/) under the accession number OneBR‐ER1.
