# Supplementary material for: Colistin‐resistant Entero bacter kobei carrying mcr‐9.1 and bla CTX‐M‐15 infecting a critically endangered franciscana dolphin (Pontoporia blainvillei), Brazil
Source: Transbound Emerg Dis. 2021 May 6;68(6):3048–54. doi: 10.1111/tbed.13980 (PMC9290994; doi:10.1111/tbed.13980)
Supplement: Supplementary file 1 — Supplementary Material [file TBED-68-3048-s001.docx]

**Supplementary Material**

**Colistin-resistant *Enterobacter kobei* carrying *mcr-9.1* and *bla*_CTX-M-15_ infecting a critically endangered franciscana dolphin (*Pontoporia blainvillei*), Brazil**


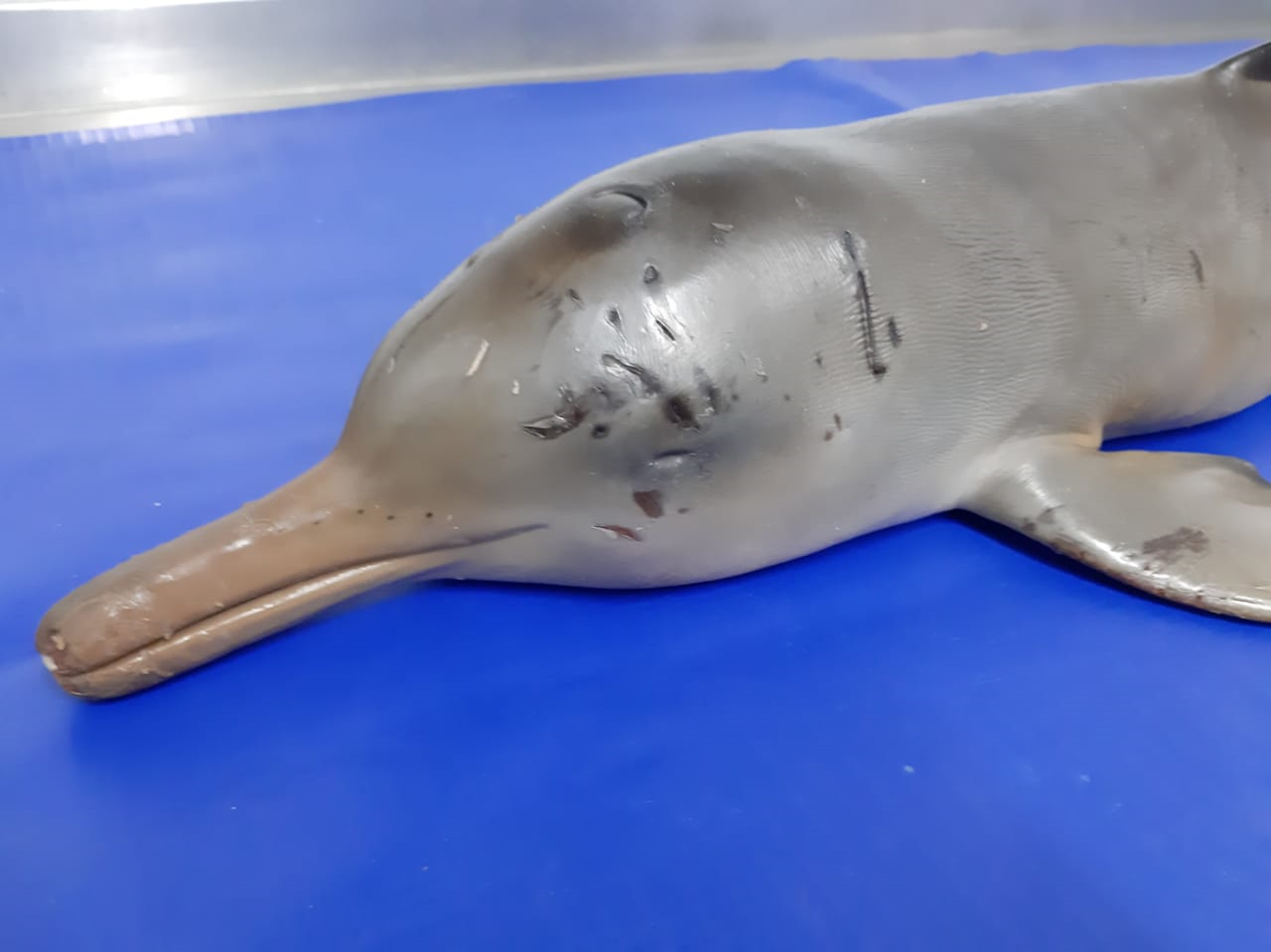


**Supplementary Figure 1.** Critically endangered franciscana dolphin (*Pontoporia blainvillei*) found in a beach, in the Southern coast of Rio de Janeiro state, Brazil. On the admission in the rescue center (Santos Basin Beach Monitoring Project), the animal presented with excoriations on the head, whereas bacteriological culture of respiratory exudate collected through the spiracle was positive for MCR-9- and CTX-M-15-produicing *Enterobacter kobei* (E11R strain).
